# Building a Bridge into the Future: Dynamic Connectionist Modeling as an Integrative Tool for Research on Intertemporal Choice

**DOI:** 10.3389/fpsyg.2012.00514

**Published:** 2012-11-20

**Authors:** Stefan Scherbaum, Maja Dshemuchadse, Thomas Goschke

**Affiliations:** ^1^Department of Psychology, Technische Universität DresdenDresden, Germany

**Keywords:** decision making, temporal discounting, intertemporal choice, date-delay effect, impulsivity, time perception, dynamic systems, connectionist modeling

## Abstract

Temporal discounting denotes the fact that individuals prefer smaller rewards delivered sooner over larger rewards delivered later, often to a higher extent than suggested by normative economical theories. In this article, we identify three lines of research studying this phenomenon which aim (i) to describe temporal discounting mathematically, (ii) to explain observed choice behavior psychologically, and (iii) to predict the influence of specific factors on intertemporal decisions. We then opt for an approach integrating postulated mechanisms and empirical findings from these three lines of research. Our approach focuses on the dynamical properties of decision processes and is based on computational modeling. We present a dynamic connectionist model of intertemporal choice focusing on the role of self-control and time framing as two central factors determining choice behavior. Results of our simulations indicate that the two influences interact with each other, and we present experimental data supporting this prediction. We conclude that computational modeling of the decision process dynamics can advance the integration of different strands of research in intertemporal choice.

## Introduction

Humans’ self-image of being a “higher” species is justified in large part by reference to our extended mental abilities. In particular, our ability to anticipate the future enables us to defy momentary temptations or impulses and to make decisions based on foresight and long-term goals (Suddendorf et al., 2009; Goschke, 2012). Conversely, we are alerted when humans appear to ignore the future consequences of their behavior. Thus, researchers have been especially interested in understanding why sometimes human choices deviate from rationality standards as defined, for instance, by the economical rule of utility maximization (Fishburn, 1968). A prominent example of such a deviation can be found in intertemporal decision making, when humans have to choose between sooner and later delivered rewards. For such decisions, the original discounted utility model prescribes that the subjective value of a delayed option should decrease as an exponential function of the time until delivery (Samuelson, 1937). In contrast to this model, empirical studies found that individuals often discount rewards more steeply, especially for small time intervals (see Frederick et al., 2002 for a review). These and other observations suggesting that human choice behavior often deviates from normative rationality standards instigated an extensive research program on intertemporal choice behavior.

Within this broad field, different lines of research can be distinguished depending on whether their primary focus is on description, explanation, or prediction. In the following, we will shortly summarize core features of these three approaches and argue for an integrative approach that combines computational modeling with experimental studies of the process dynamics of choice behavior. As an initial step, we propose dynamic connectionist modeling as a tool supporting this integration and provide a first example of its potential benefits.

The descriptive approach originated from the original discounting model (Samuelson, 1937) and has led to the development of a range of formal models proposing various mathematical functions to fit the observed temporal discounting behavior (for an overview see Doyle, 2010). Comparisons of different discounting functions including exponential, hyperbolic, and hyperbola-like functions revealed that temporal discounting is often better described by hyperbola-like functions with more than one parameter (e.g., Green et al., 1994; McKerchar et al., 2009). However, although such models carry the promise of providing precise descriptions of the outcome of intertemporal decisions, they leave open the question which information-processing mechanisms underlie the observed deviations from normative rational choice standards.

The explanatory approach aims to fill this gap and has produced a wide range of different theories which attempt to explain the general pattern of hyperbolic temporal discounting in terms of underlying cognitive mechanisms that operate at different stages of the decision process (e.g., Stewart et al., 2006; Ebert and Prelec, 2007; Killeen, 2009; Zauberman et al., 2009; Scholten and Read, 2010; Trope and Liberman, 2010). Commonly the decision process is viewed as a transformation of a sensory input into a motor output through several consecutive stages, including the stage of option representation, the stage of value representation, and the stage of the final choice (cf. Sugrue et al., 2005; Rangel et al., 2008). At the stage of option representation, hyperbolic temporal discounting has been explained by an insensitive subjective perception of prospective durations leading to a logarithmic instead of a linear perception of temporal delays (Zauberman et al., 2009). At the stage of value representation, it has been proposed that the subjective value of an option is inferred by adding the utility of a good to the disutility of a delay thus leading to hyperbolic discounting (Killeen, 2009). At the stage of the final choice, Stewart et al. (2006) proposed a continuous accumulation of a frequency count of favorable binary comparisons between the offered options and value samples retrieved from memory, with hyperbolic discounting resulting from the real-world distribution of attribute values of gains, losses, and delays. Even this exemplary set of theories shows that a multitude of plausible explanations for the hyperbolic shape of the discounting function have been proposed. This raises the question, which of the proposed mechanisms (or which combination of mechanisms) is at work in a specific decision context and which variables determine to which degree a specific decision.

The predictive approach aims to provide answers to this question and is focused on the search for specific factors influencing the result of intertemporal decisions. Amongst the multitude of possible influences, two factors gained particular attention: self-control and contextual framing (cf. Berns et al., 2007). The ability to exert self-control is assumed to reduce the extent to which behavior is determined by automatic impulses triggered by an immediately available reward (Laibson, 1997; Hofmann et al., 2009; Heatherton and Wagner, 2011). This hypothesis is supported by clinical studies showing stronger discounting in patients with disorders presumably associated with higher impulsivity such as addiction and attention deficit hyperactivity disorder (e.g., Bickel and Marsch, 2001; Wittmann and Paulus, 2008). The role of contextual framing is emphasized by findings indicating that systematic biases strongly influence the degree of discounting (e.g., Loewenstein and Prelec, 1992). For example changing the framing of the time information from delays (e.g., “in 7 days”) to calendar dates (e.g., “on the 13th of November”) reduces temporal discounting, resulting in the so-called date-delay effect (Read et al., 2005; LeBoeuf, 2006). Altogether, the empirical studies have revealed numerous contextual factors modulating and moderating intertemporal choices.

While all three strands of research reviewed so far have yielded valuable insights into intertemporal choice behavior, they have to date often been pursued relatively segregated from each other with little cross-fertilization. To further advance the understanding of mechanisms and determinants of intertemporal choice, an integration of the different empirical findings and theoretical mechanisms is needed. We therefore propose an approach, based on computational modeling and a focus on the dynamical properties of decision processes, as an approach which could offer the required integrative and explanatory power. While a dynamic, process-oriented approach is common in research on perceptual decision making (Bogacz et al., 2007; Wang, 2008), it has only recently begun to find its way into research on economic decision making where a focus on stepwise mechanisms and decision results still dominates (e.g., Summerfield and Tsetsos, 2012). However, recent empirical work demonstrates the fruitfulness of a dynamic approach. For instance, in our own recent research we investigated specific influences on temporal discounting by tracking the decision process continuously over time using a mouse tracking procedure (cf. Spivey et al., 2005; Scherbaum et al., 2011). Results indicated an interaction of the influences of self-control and contextual framing (Dshemuchadse et al., 2012): less direct choice trajectories for later/larger options indicated more reflection (i.e., enhanced self-control) in contrast to choices of the sooner/smaller options. However, this difference was reduced when time was framed in calendar dates in contrast to delays.

In the following, we aim to combine this dynamic, process-oriented approach with connectionist models, that have already demonstrated their predictive power for multiattributive choice (Roe et al., 2001; Usher and McClelland, 2001; Glöckner and Betsch, 2008; Otter et al., 2008; for a comparison of the former two models see Tsetsos et al., 2010). We will explore the potential benefit of modeling intertemporal choice within a dynamic connectionist framework in two steps. First, we develop a neural network model that integrates several of the mechanisms and influencing factors described above. This model combines a logarithmic perception of time (cf. Zauberman et al., 2009), an additive valuation process (cf. Killeen, 2009), and an accumulation process based on the statistics of our environment (cf. Stewart et al., 2006). Additionally, the model accounts for the effects of the two central factors self-control and time framing and their interaction (e.g., Wittmann and Paulus, 2008; Dshemuchadse et al., 2012). Second, we validate the proposed computational model through an empirical study exploring the interaction of the two factors self-control and time framing. In this experiment, we varied time pressure to manipulate the amount of self-control and used different framings of the time information. This way, we aimed to dissociate the influence of the two factors and test the model predictions against empirical data.

## A Computational Model of Temporal Discounting

To model intertemporal choice behavior, we implemented the process of option evaluation (e.g., Busemeyer and Townsend, 1993; Johnson and Busemeyer, 2010) in a connectionist model (see also Roe et al., 2001). In a parallel distributed network model (Rumelhart and McClelland, 1986) options are represented as different activation patterns competing with each other (e.g., Usher and McClelland, 2001; Busemeyer and Johnson, 2004). The option represented by the pattern reaching the response threshold first wins the competition and determines the final choice behavior^1^ (cf. Wang, 2008). The model incorporates the following five assumptions.

First, the activation of the option patterns accumulates gradually over time, following a non-linear activation function (cf. Usher and McClelland, 2001; Bogacz et al., 2007; Wang, 2008). The accumulation is terminated when one of the pattern reaches a threshold (cf. Busemeyer and Townsend, 1993; Wang, 2008).

Second, an option receives activation by network units representing the option attributes time interval and value (cf. Roe et al., 2001) reflecting an additive valuation process (cf. Killeen, 2009). These option attribute units represent the properties of the two options through rate coding (cf. Shadlen and Newsome, 1998; van Rullen and Thorpe, 2001). Longer time intervals are represented by less activation (and hence less support for the option), following a non-linear function as has been proposed by previous empirical work (cf. Zauberman et al., 2009). Higher values are represented by increased activation (and hence more support). Taken together, this varying activation mirrors the preference for sooner and larger options.

Third, the speed of accumulation depends on the kind of information. Specifically, we assume that time information accumulates faster than value information, leading to a general dominance of time information and hence increased temporal discounting (cf. Dshemuchadse et al., 2012). Such an increased accumulation could be the result of differences in the connection weights resulting from the statistics of our environment (cf. Stewart et al., 2006).

Fourth, the degree of self-control influences the response threshold: less self-control will lower the response threshold, thereby leading to faster responses (cf. Busemeyer et al., 2006; Wittmann and Paulus, 2008; Kim and Lee, 2011).

Five, the contextual framing of information influences the accumulation rate of information: time information presented in terms of delays accumulates faster than time information presented in terms of dates (cf. Read et al., 2005). We assume that the more complex format of calendar dates requires increased processing and therefore longer decision times in comparison to delays. This assumption is in line with similar assumptions in models of perceptual decision making, which also postulate increased processing times for more difficult stimulus material (cf. Ratcliff and Smith, 2004; Palmer et al., 2005).

To validate our model, we will analyze its discounting behavior for effects described in the literature, namely the effects of self-control and the date-delay effect. We will then report the results of a behavioral experiment based on the simulated setup and compare the empirical data to the model predictions.

## Simulation

### Model and hypotheses

In the following, we will outline the model used in our simulation (for details on the architecture and parameters see the Appendix).

#### Layers and connectivity

**Figure 1:**
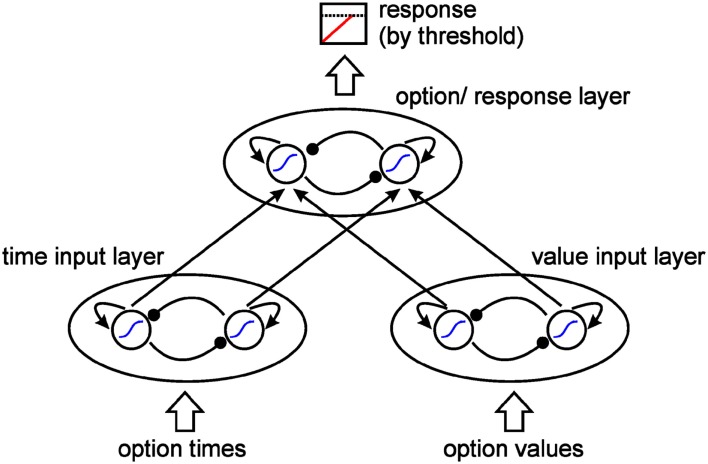
**Model architecture**. Two input layers represent times and values of the two options. A response layer represents the choice preference of the network. Each unit in the input layers excites its respective response unit. Response is elicited by reaching a response threshold. All units follow a sigmoid activation function, show recurrent excitation, and inhibit each other within each layer.

The model architecture represents a feed forward leaky competing accumulator model containing two input layers, one for time information and one for value information, and a response layer, which integrates accumulating information and indicates the tendency to choose one of the two presented options (see Figure 1, for details, please see the Appendix).

The two units within each layer inhibit each other laterally (Usher and McClelland, 2001) while activating themselves, thus supporting non-linear dynamics (Wang, 2008). Each unit within an input layer is connected to and activates the respective response unit. Hence, unit 1 in the time layer and unit 1 in the value layer both activate unit 1 in the response layer, representing option 1 (the sooner/smaller option); unit 2 in the time layer and unit 2 in the value layer both activate unit 2 in the response layer, representing option 2 (the later/larger option).

#### Activation dynamics

While layers and connections define the static architecture of the model, the unit’s activation dynamics define its reaction to an input, which is determined by the activation function. In line with previous connectionist/dynamic models, a non-linear sigmoid activation function was chosen (Cohen et al., 1992; Erlhagen and Schöner, 2002; Scherbaum et al., 2012). This ensures that each unit participates in the interaction between units only to the extent that its activation exceeds a soft threshold modeled by the sigmoid function (Erlhagen and Schöner, 2002). Hence, activation of attributes and responses and their interaction show non-linear properties. The non-linear dynamics is further enhanced by the recurrent excitatory connections, which lead to a competitive attractor dynamics (cf. Usher and McClelland, 2001; Bogacz et al., 2007; Wang, 2008).

### Simulated paradigm

We implemented an intertemporal choice task in which simulated participants had to decide which of two options they preferred: the sooner but smaller or the later but larger option.

For each participant, we orthogonally varied the interval between the options (1, 3, 5, 8, 11, and 14 days) and the value of the sooner option in percentages of the value of the later option (20, 50, 70, 85, 95, and 99%). Additionally, we orthogonally varied the time of the sooner option (0 and 7 days).

Two variables were manipulated orthogonally between simulated participants (also see the Appendix): the response threshold, simulating an impulsive (low threshold) or a self-controlled (high threshold) choice, and the timescale of accumulation for the time information, simulating the framing of the time information as dates (slower accumulation) or delays (faster accumulation). Overall, we simulated 52 participants, leading to 13 participants per condition.

At the start of each trial, two options were presented to the simulated participants. A choice was made when one of the two response units reached the response threshold.

### Data processing

To examine the amount of discounting, we determined individual discounting functions for every simulated participant in two steps. First, we identified for each block separately the indifference point, i.e., the value difference for a particular time interval where a given simulated participant chose indifferently between the two options. As an estimate of the indifference point, we determined the point of inflection of a logistic function fitted to the individual choices (sooner/smaller vs. later/larger) as a function of increasing value differences (expressed in the ration sooner/later, cf. Ballard and Knutson, 2009). In the second step, we fitted for each participant a hyperbolic function^2^ to the estimated indifference points over the different intervals and extracted the *k*-parameter of this function (Green et al., 1994).

### Results

**Figure 2:**
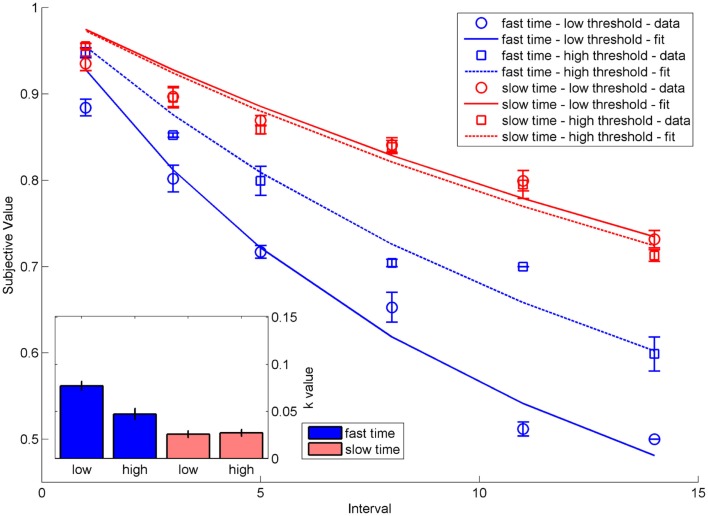
**Indifference points and hyperbolic functions depicting the decrease in subjective value as a function of intervals for the four conditions fast time accumulation – low threshold, fast time accumulation – high threshold, slow time accumulation – low threshold, slow time accumulation – high threshold**. Error bars indicate standard errors. The inset shows the *k*-values of hyperbolic functions fit to the respective data. Error bars show standard deviations.

As expected, simulated participants showed temporal discounting varying in steepness between the four different conditions (Figure 2) and varying in strength as measured by the *k*-parameter of hyperbolic functions fitted to the subjective values. Participants in the fast accumulation – low threshold condition exhibited the strongest discounting [*M*(*k*) = 0.077, SD(*k*) = 0.006], followed by participants in the fast accumulation – high threshold condition [*M*(*k*) = 0.047, SD(*k*) = 0.004]. The slow accumulation condition showed the weakest effects of discounting. Importantly, in this condition, there was no difference between the low [*M*(*k*) = 0.026, SD(*k*) = 0.003] and the high threshold condition [*M*(*k*) = 0.027, SD(*k*) = 0.004].

This indicates that the response threshold manipulation (simulating the degree of self-control) influenced the amount of discounting only when time information accumulated quickly (which by assumption is the case when time information is framed in terms of delays). The influence of the threshold vanishes when time information is accumulated slowly. However, the accumulation speed itself also influences discounting. This model behavior fits well previous empirical findings showing an interaction of the two factors self-control and contextual framing (Dshemuchadse et al., 2012).

**Figure 3:**
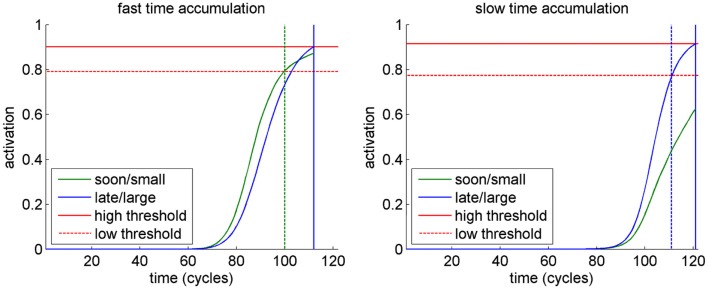
**Activation dynamics of the units in the response layer in a representative trial of one simulated participant in the different simulated conditions**. In the fast time accumulation condition (left), the activation of the sooner/smaller response dominates in beginning of a trial. Hence, lowering the threshold changes the final choices (responses indicated by vertical lines). In the slow time accumulation condition (right), there is no difference in choice between the high and the low threshold.

Looking at the activation dynamics in the response layer suggests an explanation for these results (Figure 3). If time information accumulates faster, the activation of the sooner/smaller option dominates in the first part of a trial. If the threshold is sufficiently low, this option is actually chosen. However, with a higher threshold the activation of the later/larger option catches up due to the stronger, but delayed, activation elicited by its larger value, thereby leading to a reversal of the preferred option within the trial. This difference between thresholds vanishes when time accumulates slowly, since the activation of the later/larger option dominates during the entire trial.

To corroborate this analysis, we determined the number of activation reversals within the response layer for the different conditions. For each simulated participant, we counted the number of trials in which both options were dominant at least for some time within the trial. The number of such reversal trials mirrored the observed discounting pattern, with participants in the fast accumulation – high threshold condition showing the highest number of reversals of response activation within a trial (*M* = 26.31, SD = 1.49), followed by the fast accumulation – low threshold condition (*M* = 22, SD = 1), and the two very similar slow accumulation conditions (low threshold: *M* = 4.62, SD = 1.55; high threshold: *M* = 2.92, SD = 1.5).

Hence, the higher likelihood of within-trial preference reversals in the fast accumulation – high threshold condition was associated with less discounting, as this condition offered enough time for the later but larger choice tendency to overcome a premature choice of the sooner but smaller option. In contrast to this, the low likelihood of preference reversals in the two slow accumulation conditions and the low degree of discounting were due to a dominance of the later but larger option during the entire trial.

### Discussion

As expected, we found stronger discounting in the condition simulating time framing in terms of delays (which was assumed to lead to faster accumulation of time information) compared to the condition simulating time framing in terms of dates (assumed to lead to slower accumulation of time information). Additionally, the model reproduced previous data (Dshemuchadse et al., 2012) in that it showed an effect of the simulated degree of self-control (which was implemented as a high vs. low response threshold) only in the delay condition, but not in the date condition.

An explanation for this behavior was suggested by the analysis of the activation dynamics within the response layer, which revealed reversals of the dominance of the choice options over the course of a trial (cf. Busemeyer and Townsend, 1993). In the delay condition, the faster accumulation of the time information had the effect that time information initially dominated the option preference, as it exerted a stronger influence on the activation of the option patterns than the value information. In a decision situation with low self-control – assumed to be associated with a lower response threshold – the final decision is predominantly driven by the more rapidly accumulating time information, leading to an overvaluation of time information and thus stronger temporal discounting. In contrast, in a decision situation with a higher degree of self-control – assumed to be associated with a higher response threshold – the accumulation process is prolonged, which leaves more time for the slowly accumulating value information to exert its influence on the option preferences and thus leads to less temporal discounting. In the date condition, however, this pattern changes, since by assumption the accumulation rate of the time information is reduced, leading to a more balanced influence of time and value. Therefore, in this condition the amount of temporal discounting is by and large independent from the degree of self-control.

In summary, our computational model of temporal discounting integrates theoretical assumptions derived from the explanatory approach and assumptions concerning the influence of specific factors (self-control and contextual framing) derived from a predictive approach, by specifying the non-linear dynamics of information accumulation during the option evaluation process. To validate the predictions of our model, we conducted an experiment in which we operationalized the simulated factors self-control and contextual framing and examined whether they would exert effects on human choice behavior mimicking the model predictions.

## Experiment

The aim of the experiment was to investigate in an intertemporal choice task whether the two factors self-control and contextual framing would interact in the same way as predicted by our computational model. Firstly, to manipulate the amount of self-control, we imposed a response deadline forcing subjects to respond quickly and thus severely restricting the opportunity for deliberate reflection about the choice outcomes (cf. Kim and Lee, 2011). This way we aimed to reduce the length of the accumulation process in a way comparable to a lowered response threshold or an increased initial activation of response units in connectionist models (e.g., Botvinick et al., 2001). Under these circumstances, we predicted stronger temporal discounting compared to a control condition without a response deadline. Secondly, to vary the contextual framing of time information, we capitalized on the so-called date-delay effect (Read et al., 2005; LeBoeuf, 2006), which denotes the observation that time discounting is reduced when times are presented as calendar dates instead of delays. We assumed that framing time in calendar dates would lead to slower accumulation of the time information due to the more complex format. From our simulation results we derived the prediction that framing time in calendar dates should lead to less discounting and a reduced effect of the response deadline manipulation. In summary, by independently manipulating (i) the amount of reflection and/or self-control during intertemporal choices (via imposing a response deadline) and (ii) the accumulation rate of time information (via the contextual framing), we aimed to provide empirical evidence that these two factors exert an interactive influence on temporal discounting as predicted by our model simulation.

### Materials and methods

#### Participants

Fifty students (32 female, mean age = 23.75) of the Technische Universität Dresden took part in the experiment and were assigned at random to the two framing (date vs. delay) conditions. All participants had normal or corrected to normal vision. They gave informed consent to the study and received class credit or 5 € payment.

#### Apparatus and stimuli

Stimuli were presented in white or gray on a black background on a 17 inch screen running at a resolution of 1280 × 1024 pixels (75 Hz refresh frequency). The experiment was controlled by the Eprime 1.2 software (Psychology Software Tools) running on a Windows XP SP2 personal computer. Subjects had to press the key X on a standard German computer keyboard to choose the sooner/smaller option and the key M to choose the later/larger option.

**Figure 4:**
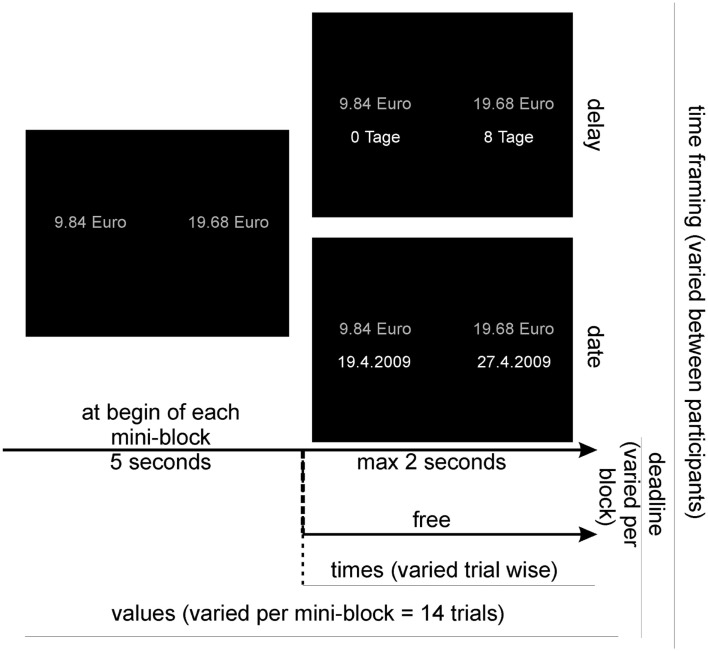
**Procedure and setup of the experiment**. The subjects were divided into two groups (varying the time framing) and the experiment was split up into blocks (varying the response deadline), consisting each of 16 mini-blocks (varying option values), consisting in turn of 14 trials (varying option delays). Before each mini-block, the option values were presented for 5 s.

Two types of screens were presented to the subjects: preparation screens and choice screens (see Figure 4). On both types of screens, the two choice options were presented on the midline of the screen, with one option on the left side (sooner and smaller option) and one option on the right side (later and larger option). The font used for the presentation was Courier New with a size of 18 points. On the preparation screen only the values of the options (e.g., “20, 23 Euro”) were presented in white color. On the choice screen, the values were presented again (albeit in gray color) and directly beneath them the corresponding delays, e.g., “in 3 Tagen” (“in 3 days”) or the corresponding dates, e.g., “19 Juli” (July, 19) were shown in white color.

#### Procedure

On each trial participants had to decide which of two options they preferred: the left (sooner but smaller) or the right (later but larger) option. They were instructed to respond to the hypothetical choices as if they were real choices. Trials were grouped into mini-blocks of 14 trials (Figure 4). For each mini-block, the two monetary values remained constant and only the times of the two options were varied. Each mini-block consisted of a preparation screen followed by 14 choice screens. The preparation screen only showed the option values and was presented for 5 s. This procedure was chosen to allow participants to encode the value information in advance, because we suspected that otherwise the amount of information especially in the response deadline condition might lead to a neglect of some of the information. However, the main goal of the response deadline was not to restrict encoding of the option information but rather to restrict the time available for subsequent reflection about the options and their anticipated future outcomes. After the preparation phase, each of the subsequent 14 choice screens additionally showed the varying option times. Each choice screen was preceded by a fixation cross presented for 500 ms. Upon the presentation of each choice screen, participants had to indicate their choice, starting 300 ms after the screen onset. In the response deadline condition, they had to respond within a time window of 1800 ms after onset of the presentation screen. If they responded too late, a feedback screen was presented indicating an error. In the control condition, no deadline was imposed and participants were free respond at any time they chose.

#### Design

The experimental design was similar to the simulated paradigm, with a slightly increased number of time intervals and value differences. Hence, for each participant and block, we orthogonally varied the time interval between the options (1, 2, 3, 5, 7, 10, and 14 days) and the value of the sooner option as percentages of the value of the later option (20, 50, 70, 80, 88, 93, 97, and 99%). The percentage of the value of the sooner option was varied between mini-blocks, while the time interval between the options was varied randomly between trials within each mini-block.

Additionally, we orthogonally varied the time of the sooner option (0 and 7 days) and the value of the later option (19.68 and 20.32 Euro). The time of the sooner option was varied to control for effects that may be specific for decisions where one of the options is immediately available (i.e., today) in contrast to decisions where both the sooner and later options are delayed. The value of the later option was varied to collect a sufficiently large number of data points without repeating identical trials, which could have induced memory effects. As neither of these two factors had any reliable effects, data was collapsed across them in the analyses reported below.

The response deadline was varied between blocks: one block with a response deadline of 1800 ms and another block without deadline were presented in random order. The framing of time (delay vs. date) was varied between subjects, who were randomly assigned to one of the two framing groups.

### Results

#### Experimental data

**Figure 5:**
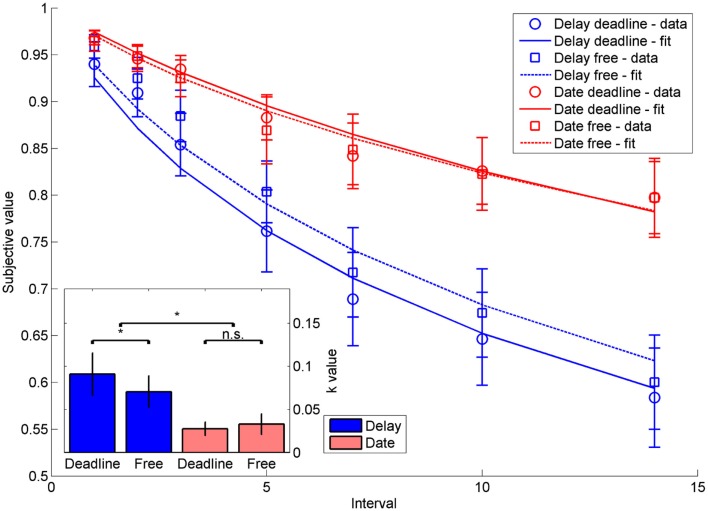
**Indifference points depicting the decrease in subjective value as a function of intervals for the four conditions delay-deadline, delay-free, date-deadline, and date-free**. Error bars indicate standard errors. The inset shows the *k*-values of hyperbolic functions fit to the respective data. Stars mark statistical significance at *p* < 0.05, error bars indicate standard errors.

On 1.62% of the trials (SD = 1.83) in the deadline condition, responses were too slow and hence not included in the analyses. As expected, participants showed varying degrees of temporal discounting in the four different conditions (Figure 5). A mixed analysis of variance (ANOVA) with the within-subjects variable response deadline (with vs. without) and the between-subjects variable time framing (delay vs. date) and the *k*-parameter of the discounting function as the dependent variable (extracted from the data analogous to the simulation data processing) revealed a significant main effect of time framing, *F*(1,48) = 4.78, *p* < 0.05, and a significant interaction between response deadline and time framing, *F*(1,48) = 7.34, *p* < 0.01. The main effect reflected steeper discounting when time was framed in terms of delays compared to dates. The interaction reflected the fact that subjects showed steeper discounting in the response deadline condition compared to the condition without deadline, but this was only the case when the time was framed in terms of delays [delays: *t*(24) = 2.12, *p* < 0.05; dates: *t*(24) = −1.16, *p* = 0.26].

To examine the effectiveness of the deadline manipulation, an analogous ANOVA was computed with response time as the dependent variable. This analysis revealed a main effect of response deadline as the only reliable result, *F*(1,48) = 25.518, *p* < 0.001 (all other *p*s > 0.3), indicating faster responses when subjects had to respond within the deadline (*M* = 760 ms, SD = 165 ms) compared to the condition without response deadline (*M* = 1013 ms, SD = 376 ms).

#### Comparison with the model predictions

To compare the simulated and experimental data, we performed two correlation analyses on the mean indifference curves in the different conditions and on the mean *k*-values in the different conditions. As expected, the indifference curves of simulated and experimental data were highly correlated (*r*^2^ = 0.96, *p* < 0.001), as were the simulated and empirically obtained *k*-values (*r*^2^ = 0.97, *p* < 0.001). There was thus a very good fit between model and experimental data.

### Discussion

As we had predicted from our computational model, the two variables response deadline and time framing not only exerted reliable effects on temporal discounting, but the experiment also yielded the expected interaction of the two variables. First, we replicated the standard date-delay effect, which was reflected in steeper discounting when time was framed in terms of delays compared to when it was framed in terms of dates. Secondly, we found that imposing a response deadline of 1800 ms induced steeper discounting compared to when participants responded at their leisure. However, most importantly, we obtained a reliable interaction between the two variables response deadline and time framing, which reflected the fact that the effect of the response deadline was only present in the delay but not in the date condition. The experiment thus replicated the critical results of the model simulation.

Interestingly, the influence of time framing was numerically much stronger than the influence of the response deadline. At present we do not know whether this reflects a genuine difference in the relative strength of the two factors or just reflects the fact that the deadline imposed in our experiment was to lenient to produce stronger effects on choice behavior. Although the effectiveness of the deadline manipulation was demonstrated by the fact that decisions times were reliably shorter when the response deadline was imposed, it must be noted that decision times in the condition without deadline were also relatively fast and on average well below the response deadline of 1.8 s. It is thus well conceivable that a stricter deadline, which would impose more severe restrictions on subjects’ opportunity to recruit self-control would exert stronger effects on choice behavior and lead to a higher proportion of choices of sooner/smaller rewards.

Furthermore, the experimental setup differs slightly from the model concerning the presentation of the options. In the experimental setup, the values of the options are presented in advance. In contrast to this, the accumulation process for time interval and value starts simultaneously in the model. We assume that, although the values have been processed prior, the option evaluation process only starts when all information is presented. In line with a previous study (Dshemuchadse et al., 2012), our results support this assumption, since time information still dominates the final decision reflected in temporal discounting.

One general concern with computational models is the number of degrees of freedom when fitting model and empirical data due to the number of parameters that could be manipulated. It is therefore important to note that the model showed temporal discounting across a wide range of parameter configurations. Likewise, the critical effects of the response threshold and the accumulation rates were obtained across a wide range of parameter settings. Furthermore, we constrained the number of free parameters by setting several parameters such as the amount of lateral inhibition to a fixed value in all layers (for more details, see the Appendix). Last but not least it should be noted that, even though care has to be taken in choosing the parameters for a model, not every simple model will succeed in producing specific results and interaction patterns simply be fine-tuning of parameters (for further discussion see McClelland, 2009). In conclusion, the present empirical results validate core predictions derived from our computational model and indicate that different framings of time information are associated with more or less complex processing operations, which influence the accumulation rate of time information and thus the impact of this information on the option preferences particularly in the early phase of the decision process. As a result, presenting time in terms of delays increases the likelihood of choosing the sooner/smaller option, due to the stronger impact of the rapidly accumulating time information compared to the more slowly accumulating time information in the date condition.

## General Discussion

In this article, we presented a dynamic connectionist model of intertemporal choice behavior by which we attempted to integrate theoretical mechanisms derived from an explanatory approach and influencing factors (i.e., self-control and contextual framing) derived from a predictive approach. Our modeling approach builds on previous connectionist models of the process of option evaluation in multiattributive choice (Roe et al., 2001; Usher and McClelland, 2001). In our simulation of an intertemporal choice task, we modeled differences in the amount of self-control by varying response thresholds (assuming that a low response threshold – by promoting rapid decisions – reduces the likelihood that time consuming self-control processes are recruited prior to the final choice). Secondly, we modeled differences in the framing of time information (dates vs. delays) by varying the activation accumulation rates in the time input layer. The simulation yielded the typical date-delay effect: in the delay condition the model exhibited increased temporal discounting compared to the date condition. Furthermore, the simulation yielded evidence for an interaction between time framing and response threshold: a reduced response threshold (assumed to reflect less self-control) increased discounting, but this was the case only in the delay condition. This pattern was related to the frequency of re-decisions or “changes of mind” (Resulaj et al., 2009) within a trial and fits with results of a previous study, in which we used movement trajectories to investigate the time course of intertemporal decision making (Dshemuchadse et al., 2012). These model predictions were further successfully validated in a new behavioral experiment, in which we manipulated the hypothesized degree of self-control by imposing a response deadline and induced different time framings via the standard date-delay manipulation.

The present model and empirical data can be viewed as an initial proof of principle demonstrating the possible gain and feasibility of an approach to intertemporal choice, that focuses on the dynamical properties of the decision process and tests specific predictions derived from computational (e.g., connectionist) modeling. In the following, we will evaluate our dynamic, process-oriented approach, and discuss the integrative benefits in the context of the three research approaches to intertemporal choice distinguished in the introduction: the descriptive, the explanatory, and the predictive approach.

The descriptive approach provides mathematical functions to formalize central aspects of temporal discounting. This approach is integrated into our data analysis, where we fitted a hyperbolic function to the discounting curves. However, in contrast to findings indicating an optimal fit for models using functions with two or more parameters (e.g., Green et al., 1994; McKerchar et al., 2009) we choose a single-parameter hyperbola for two reasons. First, since the *k*-parameter and the hyperbolic model has been widely used in other studies of discounting (e.g., Kable and Glimcher, 2007; Ballard and Knutson, 2009), we attempted to make our results directly comparable to these studies. Second, since our primary goal was to compare model predictions with the empirical data, the single-parameter hyperbolic function offers a parsimonious characterization of discounting curves in terms of a single-parameter compared to models with several interdependent parameters. In conclusion, we capitalized on insights from the descriptive approach to derive a compact quantitative description of core aspects of decision behavior (Doyle, 2010).

The explanatory approach proposes theoretical mechanisms that apply at different stages of the decision process. Three theoretical assumptions concerning mechanisms were integrated into our computational model. First, the assumption of a logarithmic perception of time (cf. Zauberman et al., 2009) was embedded into the non-linear activation function of the network units representing the option attribute “time of delivery of a reward.” Second, an additive valuation process (cf. Killeen, 2009) was implemented by having separate networks units represent the option attributes value and time, which then activated simultaneously the respective option. Third, we assumed that the accumulation of evidence (cf. Stewart et al., 2006) resulting in the final choice occurs with varying speed depending on the type of information.

Although we incorporated several mechanisms as postulated in other theories of choice behavior, we obviously also had to ignore other assumptions of these theories as well as a wide range of alternative theories not directly relevant for our dynamic modeling approach. On the one hand, we followed a process-oriented approach stemming from perceptual decision making (Bogacz et al., 2007; Wang, 2008; Summerfield and Tsetsos, 2012). Such an approach stands in contrast to theories of intertemporal choice building on stepwise mechanisms and focusing on the result of the decision (Trope and Liberman, 2003; Killeen, 2009; Loomes, 2010). On the other hand, our computational model was based on models of multiattributive choice (Roe et al., 2001; Usher and McClelland, 2001; Otter et al., 2008) with a competition process between options at its core: options are represented by different network units that inhibit each other and the choice is determined by the unit that is more strongly activated. This assumption stands in contrast to the assumptions and mechanisms of other models. For example, Stewart et al. (2006) proposed a competition between statistical frequencies: each option is compared with samples from memory, the frequency of favorable comparisons is counted, and the option with the higher frequency count is chosen. A further comparison mechanism was proposed by Scholten and Read (2010) between attributes: the attributes of the options are compared, the difference between the attributes is weighed against each other, and the more valued option is chosen. Finally, typical brain systems approaches are based on the competition between different subsystems of the brain. Metcalfe and Mischel (1999), for example, proposed that a hot brain system usually favors the sooner/smaller option and a cool brain system favors the later/larger option. Since the two systems do interact, the dominating system determines which option is chosen.

In summary, we made an attempt to integrate several mechanisms postulated within the explanatory approach into our computational model to demonstrate the potential gains of a dynamic process-oriented modeling approach to intertemporal choice. It has to be admitted, however, that in its current form our computational model is primarily intended as a proof of principle and will have to be elaborated further to explain a wider range of findings and to examine whether and in what respects its explanatory power may supersede that of alternative models of intertemporal choice (e.g., Stewart et al., 2006; Loomes, 2010; Scholten and Read, 2010). As integrative enhancements, the interaction between the different option attributes time and value (Scholten and Read, 2010) could be implemented via inhibition between the two layers; the finding of greater discounting rates for gains than for losses (Thaler and Shefrin, 1981) could be implemented via different accumulation rates as it has been done for the different time framings; the effect of stronger discounting under memory-load (Hinson et al., 2003, but see Franco-Watkins et al., 2006) can be explained with memory-load restraining resources and hence restricting deliberate reflection comparable to the influence of time restriction.

The third general approach discussed in the introduction, that we termed the predictive approach, aims to identify factors influencing intertemporal choices. Two such factors were included into our computational model and the reported experiment: the amount self-control and the contextual framing of time information. The amount of self-control was manipulated by varying the response threshold in the model and by imposing a response deadline in the experiment. Lowering the response threshold in the model led to faster responses due to a shorter process of evidence accumulation (cf. Busemeyer et al., 2006). Alternatively, one could have varied the baseline activation level to prolong or speed up responses, which, however, leads in most cases to similar results (see, e.g., Botvinick et al., 2001 in the context of a model accounting for post-error slowing). By imposing a response deadline in the experiment, we forced subjects to respond quickly, which should likewise reduce the duration of the evidence accumulation process and is known to induce more impulsive choices (Kim and Lee, 2011). Our assumption that a lowered response threshold (as induced by a response deadline) leads to reduced self-control is consistent with the fact that these processes are time consuming and fits with evidence indicating that a lack of self-control is associated with impaired behavioral inhibition and more impulsive choices (Soubrie, 1986; Stein et al., 1993). While this relatively general use of the term self-control suffices for the purposes of the present investigation, it should be noted that self-control is a multifaceted construct (e.g., Evenden, 1999; Santisteban and Arce, 2006) allowing for alternative implementations as, for instance, in theories postulating multiple decision systems (e.g., Thaler and Shefrin, 1981; Fudenberg and Levine, 2006).

To examine the influence of contextual framing – and specifically the framing of time information – on intertemporal choice, we manipulated the accumulation rate of time information in the model and the presentation format (delay vs. calendar dates) in the experiment. The manipulation of the accumulation rate rests on the assumption that the processing of dates is more complex than the processing of delays. This should lead to different rates at which time information accumulates in the respective processing layer, in a manner analogously to what has been assumed in models of perceptual decision making (cf. Ratcliff and Smith, 2004; Palmer et al., 2005). Our manipulation of the format of the time information in the experiment relied on findings from previous studies of the date-delay effect (Read et al., 2005; LeBoeuf, 2006) and yielded findings consistent with this earlier work. Nevertheless, it should be mentioned that alternative interpretations of the date-delay effect have been proposed (Read et al., 2005; LeBoeuf, 2006). While in the present study we examined two critical factors influencing intertemporal choice – self-control and contextual framing – it is an aim for future investigations to extend the present model to account for other relevant factors (see, e.g., Frederick et al., 2002) and different forms of contextual framing (see, e.g., Kahneman and Tversky, 1984).

In summary, the present model and empirical results provide an initial demonstration of the gain and feasibility of a dynamical, process-oriented approach to intertemporal choice based on computational modeling. By combining connectionist modeling and experimental data, we obtained evidence that self-control and time framing exert interactive effects on temporal discounting, which can be accounted for by dynamic properties of the decision process, in particular, the interaction of different accumulation rates and different response thresholds.

## Conflict of Interest Statement

The authors declare that the research was conducted in the absence of any commercial or financial relationships that could be construed as a potential conflict of interest.

## Footnotes

^1^ Although the representations of the magnitudes of the value and the time interval were not specified in the network architecture, one could implement a distributed representation within a specific network layer and the value of this representation could even be learned. However, the simplistic assumption of activation strength representing the magnitude of input variables is common in modeling decision processes (e.g., Roe et al., 2001; Usher and McClelland, 2001) and should not affect critical aspects of the dynamics of the accumulation process.

^2^ The fitting of the hyperbolic function was performed by applying Matlab’s multidimensional unconstrained nonlinear minimization function to the hyperbolic function 1/(1+*k*x*) = *y*, with *x* denoting time interval, *y* denoting subjective value, and *k* denoting the discounting parameter.

## Appendix

### Model architecture

The model consists of two input layers and a response layer, with two units per layer. Activation of each unit is calculated by non-linear first order differential equations as has been done previously for patterns of neural activation (Amari, 1977; Erlhagen and Schöner, 2002). Simulated by numerical integration, results were obtained using Matlab 2006a running under Windows XP SP3. The difference equation over time *t* for the activation *u* of units in a layer had the following form:

$$\tau \dot{u}\left(t\right)=-u\left(t\right)-h+wi\cdot \sigma \left(u\left(t\right)\right)+w\cdot \text{Input(}t\text{) + }N$$

Here, τ denotes the timescale, *h* the resting level, *wi* the interaction weight within the layer (self excitation and lateral inhibition), and *w* the weight of inputs into the layer; Input defines the input into the layer, *N* denotes random noise (distributed normally with *M* = 0 and SD = 0.0025), and σ denotes a sigmoid non-linearity, mirroring neural population dynamics:

$$\sigma \left(x\right)=1/\left(1+e\left(-\beta \cdot \left(x-\alpha \right)\right)\right).$$

Hence, each unit contributes to interactions in the network only to the extent that its activation exceeds a soft threshold (Cohen et al., 1992; Erlhagen and Schöner, 2002).

Following this scheme, the equation for the input layers was:

$$\tau \dot{u}\left(t\right)=-u\left(t\right)-h+wi_{i}\cdot \sigma \left(u\left(t\right)\right)+w_{si}\cdot S\left(t\right)+N$$

Here, *wi_i_* denotes the interaction weight within the input layers, *w_si_* the weight of external inputs into the layers, *S*(*t*) represents the external stimulus input into the layer. To ensure baseline levels of activations for external stimulations for all possible inputs, inputs were defined by

$$S\left(t\right)=S_{\max}-w_{si}+S_{\text{raw}}\left(t\right)\cdot w_{si}.$$

Here, *S*_max_ denotes the maximum strength of the input signal, set to 7, *S*_raw_ denotes the signals defined by the values and times of the respective options (ranging from 0 to 1, see model input, below), and *w_si_* denotes the weight of the input with respect to *S*_max_, set to 0.3 for times and 0.7 for values.

For the input layer representing value information, we set τ*_V_* = 30. For the input layer representing time information, we simulated different speeds of information accumulation, by setting τ*_T_* = 10 for the fast accumulation condition and setting τ = 30 for the slow accumulation condition.

Analogously to the input layers, the equation for the response layer was:

$$\tau \dot{u}\left(t\right)=-u\left(t\right)-h+wi_{r}\cdot \sigma \left(u\left(t\right)\right)+w_{i_{1}r}\cdot I_{1}\left(t\right)+w_{i_{2}r}\cdot I_{2}\left(t\right)+N$$

Here, *wi_r_* denotes the interaction weight within the response layer,$w_{i_{1}r}$ $w_{i_{2}r}$ denote the strength of input from the input layers, and *I*_1_ and *I*_2_ represent the signal from the input layers. Responses were considered as made when σ (*u*(*t*)) reached a response threshold. This threshold was sampled at random from a normal distribution with an SD of 0.0075 and a mean of 0.9 for the high threshold condition and a mean of 0.77 in the low threshold condition. The timescale of information accumulation was set to the same value as for the value input layer, τ*_V_*, hence τ = 30.

The weight matrices are shown in the following. The interactions within the input layers, and the response layer were defined by

$$wi_{i}=\left(\;\begin{array}{cc} 1 & -2\\ -2 & 1 \end{array}\right),.\;wi_{r}=\left(\;\begin{array}{cc} 1 & -2\\ -2 & 1 \end{array}\right)$$

Hence, within all layers, there was the same strong lateral inhibition compared to a weaker self excitation of each node.

Signal transmission from each input layer to the response layer was defined by

$$w_{i_{1}r}=w_{i_{2}r}=\left(\;\begin{array}{cc} 5 & 0\\ 0 & 5 \end{array}\right).$$

Hence, the input layers were associated equally with the response layer and each unit within an input layer representing the time or value of an option activated the response unit representing the preference for this option.

The other parameters where chosen as follows: *h* = 5, α = 0, β = 1.5.

The parameters *h*, α, β, *S*_max_ and the connection weights *w_si_*, *wi_i_*, *wi_r_*,$w_{i_{1}r}$ $w_{i_{2}r}$ were chosen to produce classical discounting behavior. By choosing equal values for *wi_i_* and *wi_r_* as well as for $w_{i_{1}r}$ $w_{i_{2}r}$ we aimed to minimize the number of free parameters and keep the model as simple as possible. Within these constraints, the model’s discounting behavior was qualitatively similar across a wide range of parameter choices.

The two critical parameter variations concerned the response threshold (0.9 vs. 0.77) and the accumulation rate of the time information τ*_T_*. Again, we set τ*_T_*, τ*_R_*, and τ*_T_* to equal values (in the slow accumulation condition) to minimize the number of free parameters. Hence, the only free parameter was τ*_T_* in the fast accumulation condition, with the constraint τ*_T(fast)_* < τ*_T(fast)_*. Within these constraints, the presented effects were qualitatively stable across a wide range of parameter combinations.

### Calculation of model input and procedure

The input to the input layers representing time and value information for options 1 and 2 was defined by the input vectors *S_T_* = (*T*_1_,*T*_2_) and *S_V_* = (*V*_1_,*V*_2_). *S_T_* and *S_V_* were varied orthogonally (see description of paradigm). For *S_T_*, *T*_1_ was chosen from {0,7}. *T*_2_ was defined by *T*_1_ + *T_I_*, the interval between the options, with *T_I_* chosen from {1, 3, 5, 8, 11, 14}. *S_T_* was then transformed to normalized input values by

$$S_{T}=1-\sqrt{S_{T}/T_{\max}}.$$

Hence, time was normalized to the maximum possible time value, transformed non-linearly to mirror non-linear time perception (see main text) and inverted, so that lower times lead to higher input activation, mirroring the preference for smaller delays.

For *S_V_*, *V*_2_ was set to 1, and *V*_1_ was chosen so that the ration *V*_1_/*V*_2_ was {0.2, 0.5, 0.7, 0.85, 0.95, 1}. Since *S_V_* was already normalized to a maximum value of 1, no further transformation was necessary to receive normalized input values. Hence, higher values lead to higher input activation, mirroring the preference for high values.

Each trial began with an inter-trial interval of 50 cycles without input, followed by the activation of the input vectors. The trial ended when the output activation of one of the two response units reached the response threshold and, hence, a choice was performed.
